# Tibial implant migration of cemented and cementless total and unicompartmental knee arthroplasties in patients with low and normal bone mineral density: a 5-year RSA cohort study of 397 cases

**DOI:** 10.2340/17453674.2026.46362

**Published:** 2026-08-10

**Authors:** Karina N LINDE, Bente L LANGDAHL, Søren RYTTER, Rob G H H NELISSEN, Maiken STILLING

**Affiliations:** 1 Department of Orthopaedics, Aarhus University Hospital, Aarhus, Denmark; 2 AutoRSA research group, Aarhus University Hospital, Aarhus, Denmark; 3 Department of Clinical Medicine, Aarhus University, Aarhus, Denmark; 4 Department of Endocrinology and Internal Medicine, Aarhus University Hospital, Aarhus, Denmark; 5 Department of Orthopedics, Leiden University Medical Center, Leiden, The Netherlands

## Abstract

**Background and purpose:**

Periprosthetic bone may influence the longevity of joint implants. We aimed to investigate the association between bone mineral density (BMD) and tibial implant migration of cemented and cementless knee arthroplasty as a surrogate marker of aseptic loosening.

**Methods:**

In a prospective cohort study, patients were operated on between 2014 and 2018 with a unicompartmental knee arthroplasty (UKA) or a total knee arthroplasty (TKA). Preoperative BMD was measured using dual-energy X-ray absorptiometry of the lumbar spine and hips. Patients were categorized into low or normal T-score groups (threshold: T-score ≤ –1.0). Postoperative tibial implant migration was assessed using radiostereometry at baseline and 1-, 2-, and 5-year follow-ups. The primary outcome was 1-year maximum total point motion (MTPM) differences between T-score groups. Secondary outcomes evaluated the association between continuous migration (MTPM > 0.2 mm between 1 and 2 years) and T-score.

**Results:**

397 patients were included, where 210 patients received a cementless implant (TKA = 78, UKA = 132) and 187 received a cemented implant (TKA = 83, UKA =104). Estimated 1-year mean MTPM differences between the low and normal T-score groups were 0.15 mm (CI −0.25 to 0.55) for cementless TKA, 0.12 mm (CI −0.25 to 0.49) for cemented TKA, −0.21 mm (CI −0.51 to 0.10) for cementless UKA, and −0.15 mm (CI −0.34 to 0.04) for cemented UKA. A 1-unit increase in T-score showed comparable odds of continuous migration within both cemented (OR 0.94, CI 0.68–1.30) and cementless (OR 0.79, CI 0.56–1.12) tibial implants.

**Conclusion:**

Tibial implant migration was not significantly different between patients with low and normal BMD as defined by T-score groups. These findings should be interpreted cautiously due to small group sizes and wide confidence intervals, highlighting the need for further studies to clarify the role of BMD in implant migration.

Osteoporosis, characterized by low bone mineral density (BMD), is common in patients with knee osteoarthritis (OA) scheduled for knee arthroplasty [1,2]. Low BMD compromises the skeletal regenerative capacity, which may lead to increased bone resorption at the bone–implant interface and impaired implant osseointegration and fixation, thereby increasing the risk of aseptic loosening [3]. In line with this, studies have reported that osteoporosis negatively affects the outcomes of total knee arthroplasty (TKA) with increased risks of periprosthetic fractures, prosthetic joint infection, aseptic loosening, and revision [4-6]. Higher early implant migration strongly correlates with an increased risk of implant loosening and subsequent revision surgery at long-term follow-up measured by radiostereometric analysis (RSA) [7,8]. Thus, early implant migration allows for the investigation of risk factors associated with inferior fixation at long-term follow-up. The association between preoperative BMD and tibial implant migration has been previously assessed in smaller-scale studies (n < 100), yielding conflicting results [9-14]. Thus, evidence for surgical guidelines regarding the use of cemented or cementless tibial implant fixation methods in patients with osteopenia/osteoporosis vs normal BMD is still warranted.

We aimed to investigate the association between preoperative BMD at the lumbar spine and total hip and tibial implant migration in cemented and cementless TKA and unicompartmental knee arthroplasty (UKA). We hypothesized that patients with low BMD had a higher early tibial implant migration (1-year maximum total point motion [MTPM]) than patients with normal BMD.

## Methods

### Study design

This prospective clinical cohort study included an unselected patient cohort with knee OA scheduled for TKA or UKA at Aarhus University Hospital between 2014 and 2018 (AutoRSA database) [12,15]. The study is reported according to STROBE guidelines. Patients were examined preoperatively with dual-energy X-ray absorptiometry (DXA) of the lumbar spine and total hips. Postoperatively, patients underwent evaluation of tibial implant migration using RSA [16] immediately after surgery and at 1-, 2-, and 5-year follow-ups. The inclusion criteria were a preoperative DXA and minimum 2 RSA images available (1 baseline and 1 follow-up image). In bilateral cases, data from the second knee was excluded.

### Surgery

**Figure 1:**
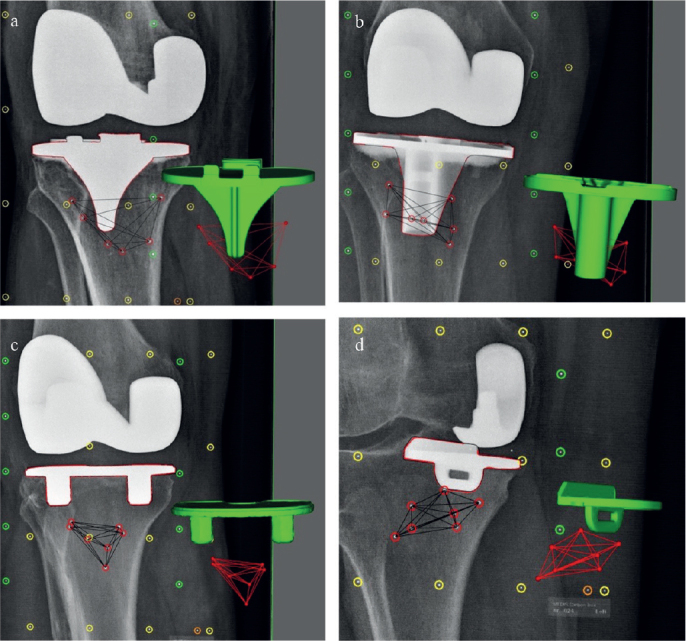
Postoperative RSA images of the 5 implants included: (a) Cemented Vanguard TKA with a finned stem, (b) cemented NexGen stemmed precoat TKA, (c) cementless NexGen Monoblock TKA, and (d) cemented or cementless Oxford UKA. RSA: radiostereometric analysis, TKA: total knee arthroplasty, UKA: unicompartmental knee arthroplasty.

All patients underwent surgery at Aarhus University Hospital by 5 experienced knee surgeons. The surgical procedures followed the recommendations provided by the arthroplasty manufacturers. For this study 5 implant types were included: cemented Vanguard TKA (Zimmer Biomet) n = 12, cemented NexGen Stemmed Precoat TKA (Zimmer Biomet) n = 71, cementless NexGen Monoblock TKA (Zimmer Biomet) n = 78, cemented Oxford UKA (Zimmer Biomet) n = 104, and cementless Oxford UKA (Zimmer Biomet) n = 132 (Figure 1). The criteria for using cementless implants were age below 75 years and good bone quality (i.e., assessed at preoperative radiographs and intraoperative evaluation by the surgeon). During surgery, 1.0 mm tantalum beads (n = 3–8) were inserted into the proximal tibia, necessary for RSA implant migration measurements.

### Radiostereometric analysis (RSA)

The RSA guidelines were followed [16]. Baseline RSA images were obtained within 2 days postoperatively after mobilization, allowing full weightbearing. Follow-up RSA images were performed at 1-, 2-, and 5-years postoperatively. A standardized RSA setup was used [12]. 2 experienced observers analyzed RSA images using Model-Based RSA version 4.2015 software (RSAcore, Leiden, The Netherlands). The baseline RSA served as the reference. The coordinate system of the calibration box was utilized. Double examinations were performed at 1 year to assess RSA precision [16]. If the MTPM difference between the double examinations was > 0.5 mm, the RSA analyses were reanalyzed to correct potential errors. Before statistical analyses, implant migration data was visually inspected using individual scatterplots. The upper limit for the mean error of rigid-body fitting (ME) was 0.35 and for the condition number (CN) it was 120. Implant migration was evaluated by maximum total point motion (MTPM), translation (Tx, Ty, Tz, total translation [TT]), and rotation (Rx, Ry, Rz, total translation [TR]). Furthermore, implant migration was defined as continuous migration, with an MTPM > 0.2 mm between 1- and 2-year follow-ups, which has been shown to indicate that implants are at risk of tibial implant loosening at 10 years with a predictive power of 85% (8). Additionally, continuous migration between 2 and 5 years with MTPM > 0.1 mm/year was also reported, as this strengthens the prediction of this cut-off value [8].

RSA precision measured at 1-year follow-up (n = 365) is presented in Table S1 (see Supplementary data). For 9 patients, RSA analyses were reanalyzed due to MTPM precision difference > 0.5 mm.

### Dual-energy X-ray absorptiometry

Preoperative BMD was measured using DXA of the lumbar spine and bilateral total hip (GE Medical Systems, Lunar iDXA, Madison, WI, USA, Encore software version 16). Patients diagnosed with osteoporosis (T-score ≤ –2.5) were referred to the local department of endocrinology for evaluation of the need for treatment. No patients were excluded based on their T-score. BMD was assessed using 3 measurement sites (lumbar spine, right total hip, and left total hip) and by the lowest T-score from the 3 sites.

### Sample size

The primary outcome measure was tibial implant migration evaluated by 1-year MTPM. For the cementless groups, with a minimum of 28 patients in each group, power of 80%, a significance level of 0.05, and an SD of 0.7 mm, an a priori calculation performed before conducting the statistical analyses resulted in a minimal detectable difference of 0.54 mm between groups [7,17]. For the cemented groups, with a minimum of 25 patients in each group, a power of 80%, a significance level of 0.05, and an SD of 0.4 mm, the minimal detectable difference was 0.32 mm [17].

### Statistics

The primary outcome was the 1-year MTPM in T-score groups, where patients were categorized based on the lowest T-score: normal T-score (T-score > –1.0) and low T-score (T-score ≤ –1.0: osteopenia and osteoporosis as defined by World Health Organization). The primary analyses were performed separately for the cemented TKA group, the cementless TKA group, the cemented UKA group, and the cementless UKA group.

A linear mixed-effect model for repeated measures was used to account for repeated measurements within patients. MTPM was included as the dependent variable, and T-score group, time (1, 2, and 5 years), and their interaction were included as fixed effects. Time was modelled as a categorical variable to allow estimation of between-group differences at each follow-up time-point. The models were adjusted for age and body mass index (BMI).

A random intercept for patient was included to account for within-patient correlation, and an unstructured covariance matrix was applied to model residual correlations over time. Parameters were estimated using restricted maximum likelihood. Model-based marginal means (least-square means) were estimated for each group at each time-point, and between-group differences with 95% confidence intervals (CI) were derived from the fitted model. The primary effect measure was the between-group difference in MTPM at 1 year, obtained from the group-by-time interaction. Missing data was assumed to be missing at random, and the mixed-effects model uses all available observations under a likelihood-based framework. As a sensitivity analysis, complete-case analyses were performed. Model assumptions were assessed by inspection of Q–Q plots of residuals and plots of residuals vs fitted values, which indicated no major deviations from normality or homoscedasticity. No influential outliers were identified. To assess the potential influence of antiresorptive treatment on the association between T-score and MTPM, the analyses were repeated after exclusion of patients receiving antiresorptive treatment.

A secondary analysis was performed using logistic regression models to estimate the odds ratio (OR) for an MTPM > 0.2 mm between 1 and 2 years of follow-up [8], using the preoperative T-score as a continuous variable. Linear regression models were conducted to examine the association between the preoperative T-score and MTPM between 1 and 2 years of follow-up as a continuous variable. These analyses were performed to provide additional insight into the association between BMD and implant migration by modelling T-score as a continuous exposure. As the 0.2 mm threshold is applicable to both UKA and TKA, the models were stratified by fixation only.

As exploratory analyses, the associations between 1-year MTPM and subsidence, and preoperative BMD were analyzed using 3 different multivariate linear regression models. Each model included 1 of the following BMD measures: the lowest BMD in the 3 measurement sites (lumbar spine, total right hip, or total left hip), lumbar spine BMD, or mean total hip BMD. All models reported the regression coefficient (β).

Models using T-score were BMI and age-adjusted, and models using BMD were adjusted for BMI, sex, and age.

For baseline characteristics means and proportions were calculated.

The statistical level of significance was P < 0.05 (2-sided). Stata IC version 19.5 (StataCorp, College Station, TX, USA) was used for statistical analyses.

### Ethics, registration, use of AI tools, funding, and disclosures

The study was conducted in accordance with the Helsinki II declaration, and patients gave informed oral consent. The AutoRSA database and the study were approved by the National Ethics Committee (no. 2302161 issued March 27, 2023) and the Danish Data Protection Agency (1-16-02-54-14, issued on February 3, 2014). Data was managed using the REDCap electronic data capture tools hosted at Aarhus University [18]. ChatGPT (OpenAI) was used for grammar correction and language editing to improve clarity and readability of the manuscript. The authors reviewed and revised all AI-assisted edits and take full responsibility for the final content of the manuscript, including the interpretation of data and scientific conclusions.

Funding for the study: Karen Elise Jensen’s Foundation, Health Research Foundation of Central Denmark Region, A.P. Moller Foundation, Danish National Advanced Technology Foundation, the Development Fund at Aarhus University Hospital, Orthopedic Research Foundation in Aarhus, and Aase and Ejnar Danielsens Foundation. The funding parties did not take part in the planning, execution, or interpretation of this study. Complete disclosure of interest forms according to ICMJE are available on the article page, doi: 10.2340/17453674.2026.46362

## Results

**Figure 2:**
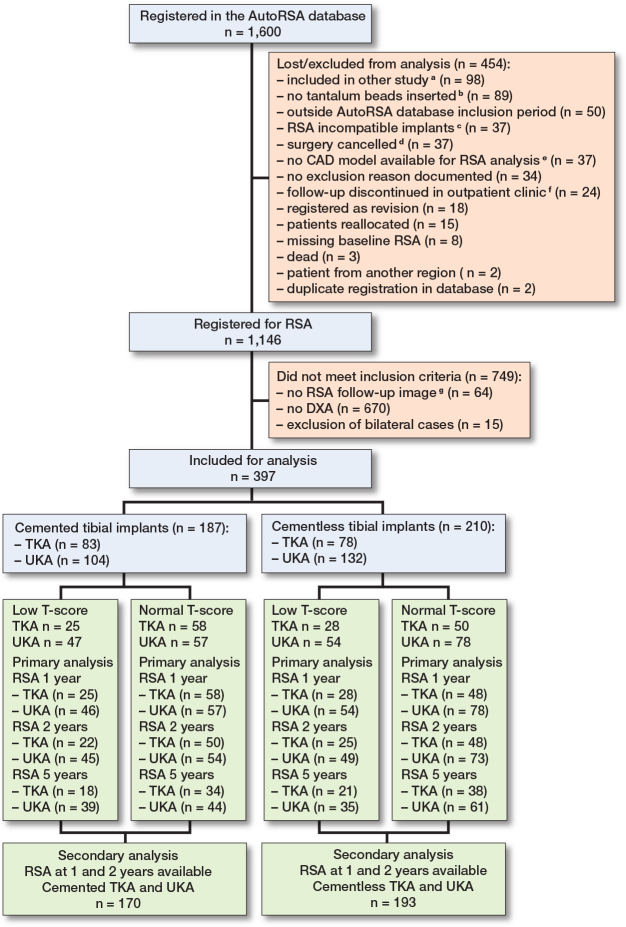
Patient flowchart. **^a^** Trial with adjuvant antiresorptive treatment at the same institution. **^b^** Bead gun unavailable or unsterile, no beads inserted, bone quality too poor, patient declined, or beads were dropped on the floor. **^c^** Patellofemoral prostheses or Legacy constrained condylar knee. **^d^** Due to comorbidity or other illness or no longer knee pain. **^e^** Lateral UKA, NexGen rotating Hinge or NexGen Wedged. **^f^** Language barriers, unable to attend follow-ups or residing far away. **^g^** Death (n = 18), discontinued in outpatient clinic (n = 20), revised (n = 16), unknown reasons (n = 5), RSA image not suitable for analyses (n = 4); poor distribution, no beads visible, loosening of beads. RSA 1 year: 3 RSA images were missing due to non-attendance, incomplete data collection, or technical errors. RSA 2 years: 31 RSA images were missing (11 due to death, 4 due to cancellation performed by the patients, 5 due to revision, 13 due to non-attendance, incomplete data collection, or technical errors). RSA 5 years: 107 RSA images were missing (25 due to death, 10 due to cancellation performed by the patients, 7 due to revision, 65 due to non-attendance, incomplete data collection, or technical errors) CAD: computer-aided design, DXA: Dual-energy X-ray absorptiometry RSA: radiostereometric analysis, TKA: total knee arthroplasty, UKA: unicompartmental knee arthroplasty.

397 patients met the inclusion criteria. The excluded 64 patients with only 1 RSA image were older than the total cohort (mean difference: 3.4 years, CI 0.9–5.9, P = 0.001) (Figure 2). In the period from inclusion to the study endpoint, 7 implants were revised, and 25 patients died. Baseline characteristics are described in Tables 1 and 2.

**Table 1:** Baseline characteristics stratified by implant type and T-score group

Characteristic	T-score group		Total
	Low ^a^	Normal	
Cementless TKA, n	28	50	78
Age, mean (SD)	65 (10)	62 (9.9)	63 (10)
BMI, mean (SD)	28 (5.6)	30 (4.7)	29 (5.1)
Women, n (%)	19 (68)	23 (46)	42 (54)
Cementless UKA, n	54	78	132
Age, mean (SD)	70 (9.0)	66 (9.1)	68 (9.2)
BMI, mean (SD)	27 (3.9)	30 (4.5)	29 (4.5)
Women, n (%)	35 (65)	35 (45)	70 (53)
Cemented TKA, n	58	25	83
Age, mean (SD)	76 (7.4)	74 (9.1)	75 (8.0)
BMI, mean (SD)	28 (4.9)	32 (6.3)	29 (5.6)
Women, n (%)	47 (81)	17 (68)	64 (77)
Cemented UKA, n	47	57	104
Age, mean (SD)	67 (8.3)	64 (9.5)	66 (9.1)
BMI, mean (SD)	28 (4.4)	29 (4.4)	28 (4.4)
Women, n (%)	28 (60)	24 (42)	52 (50)

a Low = patients with T-score ≤ –1.0.

BMI: body mass index, SD: standard deviation, TKA: total knee arthroplasty, UKA: unicompartmental knee arthroplasty.

**Table 2:** T-score and BMD measurements in the patient population

Factor	Low T-score group		Normal T-score group ^c^	Mean BMD (SD)
	Osteo-porosis ^a^	Osteo-penia ^b^		
Cementless TKA	5	23	50	0.98 (0.14)
Osteoporosis treatment	4	0	0	
Cementless UKA	9	45	78	0.98 (0.16)
Osteoporosis treatment	6	5	2	
Cemented TKA	13	45	25	0.89 (0.17)
Osteoporosis treatment	11	5	3	
Cemented UKA	10	37	57	0.98 (0.15)
Osteoporosis treatment	5	5	0	
Total	37	150	210	
Osteoporosis treatment	26	15	5	

a T-score ≤ –2.5

b T-score ≤ –1.0 and > –2.5

c T-score > –1.0

For abbreviations, see Table 1.

### Primary outcome

**Figure 3:**
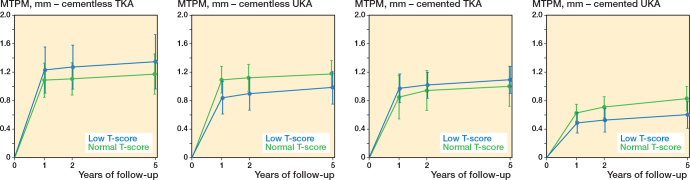
Mean MTPM (CI) in the low and normal T-score groups in cementless TKA (n = 78), cementless UKA (n = 132), cemented TKA (n = 83), and cemented UKA (n = 104). The low T-score group includes patients with T-score ≤ –1.0. MTMP: maximum total point motion, TKA: total knee arthroplasty, UKA: unicompartmental knee arthroplasty.

Similar mean MTPM values were found throughout follow-up between T-score groups for cementless TKA, cementless UKA, cemented TKA, and cemented UKA (Figure 3A–D, Table 3). Patients with higher T-score had a little less migration but not statistically significantly in both cemented and cementless TKA. In UKA a reverse migration pattern was shown. Sensitivity analyses excluding patients receiving antiresorptive treatment (4 cementless TKA, 13 cementless UKA, 19 cemented TKA, and 10 cemented UKA) did not alter the findings. Translation and rotation are presented in Tables S2–S5 (see Supplementary data).

**Table 3:** Comparison of mean MTPM (CI) between low and normal T-score groups in millimeters adjusted for BMI and age

Implant	Low T-score ^a^, mean MTPM (CI)	Normal T-score, mean MTPM (CI)	Difference in MTPM (CI)
Follow-up			
Cementless TKA, n	28	50	
1 year	1.23 (0.91–1.55)	1.09 (0.85–1.33)	0.14 (–0.26 to 0.55)
2 years	1.27 (0.96–1.58)	1.11 (0.88–1.33)	0.17 (–0.22 to 0.55)
5 years	1.35 (0.96–1.73)	1.17 (0.89–1.46)	0.17 (–0.30 to 0.65)
Cementless UKA, n	54	78	
1 year	0.84 (0.61–1.07)	1.10 (0.91–1.29)	–0.26 (–0.56 to 0.05)
2 years	0.90 (0.67–1.12)	1.12 (0.94–1.31)	–0.23 (–0.53 to 0.08)
5 years	0.98 (0.75–1.21)	1.18 (0.99–1.37)	–0.20 (–0.50 to 0.11)
Cemented TKA, n	58	25	
1 year	0.97 (0.77–1.17)	0.84 (0.54–1.15)	0.12 (–0.25 to 0.50)
2 years	1.01 (0.83–1.19)	0.94 (0.66–1.22)	0.07 (–0.27 to 0.41)
5 years	1.09 (0.90–1.27)	1.00 (0.72–1.28)	0.09 (–0.25 to 0.43)
Cemented UKA, n	47	57	
1 year	0.49 (0.35–0.63)	0.62 (0.49–0.75)	–0.13 (–0.33 to 0.06)
2 years	0.52 (0.36–0.69)	0.71 (0.56–0.86)	–0.18 (–0.41 to 0.04)
5 years	0.60 (0.41–0.79)	0.83 (0.66–1.00)	–0.23 (–0.49 to 0.03)

a The low T-score group includes patients with T-score ≤ –1.0.

For abbreviations, see Table 1 and CI: 95% confidence interval, MTPM maximum total point motion.

Complete-case analyses included 177 observations compared with 234 in the primary analysis for the cementless TKA group. In this group, point estimates differed in direction at 1, 2, and 5 years compared with the primary analysis, although CIs overlapped. As an additional sensitivity analysis, the complete-case analyses were repeated, excluding the 5-year follow-up due to increased missing data (Figure 2), including 213 observations. Results were consistent with the primary analysis at 1 and 2 years. In the remaining 3 implant groups, results were consistent between the primary and complete-case analyses.

### Secondary outcomes

#### Continuous migration

Among cementless implants, 32 implants showed continuous migration (MTPM > 0.2 mm between 1 and 2 years), whereas 161 implants did not (Tables 4 and 5). Among cemented implants, 34 implants showed continuous migration (MTPM > 0.2 mm between 1 and 2 years) and 136 implants did not. A 1-unit increase in preoperative T-score showed comparable odds of continuous migration within both cemented (OR 0.96, CI 0.68–1.30) and cementless (OR 0.79, CI 0.56–1.12) tibial implants.

**Table 4:** Association between preoperative T-score and MTPM > 0.2 mm between 1 and 2 years presented as OR and CI adjusted for BMI and age

Cohort	Preoperative T-score vs MTPM > 0.2 mm between 1 and 2 years follow-up, logistic regression				
TKA and UKA	n	Crude OR (CI)	P value	Adjusted ^a^ OR (CI)	P value
Cementless	193	0.77 (0.55–1.07)	0.1	0.79 (0.56–1.12)	0.2
Cemented	170	0.99 (0.74–1.34)	>0.9	0.94 (0.68–1.30)	0.7

a Logistic regression models were adjusted for BMI and age.

For abbreviations, see Table 1 and CI: 95% confidence interval, MTPM: maximum total point motion, OR: odds ratio.

**Table 5:** Association between preoperative T-score and change in MTPM (mm) between 1 and 2 years’ follow-up presented as regression coefficient (β) in millimeters with CI adjusted for BMI and age

Cohort	Preoperative T-score vs MTPM between 1 and 2 years’ follow-up, linear regression						
TKA and UKA	n	Crude β (CI)	R^2^	P value	Adjusted ^a^ β (CI)	R^2^	P value
Cementless	193	–0.02 (–0.05 to 0.00)	0.01	0.1	–0.02 (–0.05 to 0.01)	0.02	0.2
Cemented	170	0.03 (–0.03 to 0.08)	0.01	0.3	0.03 (–0.02 to 0.09)	0.02	0.3

a Linear regression models were adjusted for BMI and age.

For abbreviations, see Table 1 and CI: 95% confidence interval, MTPM: maximum total point motion, R^2^ = coefficient of determination.

The total number of implants with continuous migration is shown in Table S6 (see Supplementary data).

### Exploratory analyses

In a sub-analysis, similar associations were found between preoperative BMD and 1-year MTPM or subsidence (Ty) for all implant subgroups (Table 6).

**Table 6:** Association between 1-year mean MTPM (mm), 1-year mean subsidence (mm), and preoperative BMD presented as regression coefficient (β) in millimeters with CI adjusted for BMI, sex, and age

Implant	Lowest BMD ^a^		Lumbar spine BMD		Mean total hip BMD ^b^	
1-year migration	β (CI)	R²	β (CI)	R²	β (CI)	R²
Cementless TKA						
MTPM	–0.42 (–1.96 to 1.13)	0.06	–0.81 (–2.13 to 0.51)	0.08	–0.46 (–1.99 to 1.07)	0.06
Subsidence	0.14 (–0.47 to 0.75)	0.05	0.51 (–0.04 to 1.05)	0.16	0.10 (–0.50 to 0.71)	0.05
Cementless UKA						
MTPM	0.16 (–0.86 to 1.19)	0.03	0.17 (–0.91 to 1.25)	0.06	0.53 (–0.63 to 1.66)	0.03
Subsidence	–0.19 (–0.65 to 0.26)	0.02	–0.03 (–0.47 to 0.40)	0.05	–0.32 (–0.84 to 0.19)	0.03
Cemented TKA						
MTPM	–0.84 (–2.0 to 0.34)	0.08	–0.25 (–0.96 to 0.47)	0.05	–1.27 (–2.77 to 0.22)	0.13
Subsidence	0.08 (–0.19 to 0.35)	0.04	0.04 (–0.20 to 0.27)	0.05	0.14 (–0.20 to 0.48)	0.06
Cemented UKA						
MTPM	–0.43 (–1.13 to 0.27)	0.14	0.05 (–0.43 to 0.53)	0.13	–0.18 (–1.00 to 0.63)	0.12
Subsidence	–0.20 (–0.60 to 0.19)	0.02	–0.08 (–0.26 to 0.11)	0.06	–0.27 (–0.73 to 0.18)	0.03

a Lowest measured BMD of either the lumbar spine or total hips.

b Average of left and right total hip.

For abbreviations, see Table 1 and CI: 95% confidence interval, MTPM: maximum total point motion, R^2^ = coefficient of determination.

#### RSA analyses and precision

In the analyzed patient cohort (n = 397), ME at 1 year was 0.18 (SD 0.07 range 0.03–0.35). At 5 years, 1 patient had an ME > 0.35 (ME = 0.37) due to a small marker model, the ME was accepted, and the data was included (CN = 87). The mean CN was 59 (SD 33, range 23–471). 14 patients had a CN >120 but they had 3–7 markers, acceptable migration patterns, and a mean 1-year ME of 0.22 (SD 0.08). The data was included. Sensitivity analyses excluding patients with CN >120 did not alter the conclusions.

## Discussion

We aimed to investigate the association between BMD and tibial implant migration as a surrogate marker of aseptic loosening. We found similar MTPM between low and normal T-score groups for all implant subtypes. Furthermore, a 1-unit increase in preoperative T-score showed comparable odds of continuous migration within both cemented and cementless tibial implants. Likewise, similar associations were observed between the lowest BMD of the lumbar spine or total hips and 1-year MTPM and subsidence of the tibial implants.

Our study showed that, for TKA, the mean MTPM was higher in the low T-score group than in the normal T-score group, although this difference did not reach statistical significance with overlapping CIs. Similarly, the regression analyses did not demonstrate statistically significant associations between BMD and 1-year MTPM or subsidence; however, the regression coefficients suggested less migration with higher BMD for both cemented and cementless TKA. In contrast, UKA demonstrated a reverse migration pattern. These findings may indicate that factors other than lumbar spine or total hip BMD play a more prominent role in UKA migration, such as the amount of (cortical vs spongious) bone coverage of the tibial implant. This could also explain why studies have shown that cementless UKA was safe in patients with reduced BMD [19], and had similar revision rates to cemented UKAs in patients > 70 years of age [20]. However, local tibial BMD may still be important for cementless UKA fixation. In medial knee OA, varus malalignment induces loading-related adaptations that preserve BMD in the medial compartment, thereby potentially ensuring adequate local bone conditions for cementless fixation in older or osteoporotic patients.

Although the preoperative T-score showed similar associations with continuous migration for any implant subtype in our study, the findings for cementless implants suggested lower odds of continuous migration with a higher T-score but did not reach significance. This was indicated by the mean effect (OR 0.79) and CI (ranging from a potential 44% decrease to a 12% increase in the odds). Other studies have found an association between less continuous migration and a higher preoperative tibial BMD for cementless TKA and not for cemented TKA [9,14].

The general hypothesis that patients with low BMD have a higher implant migration could not be supported by our study. The reason may be due to the absence of a true association between BMD of the lumbar spine and total hip and implant migration, patient selection, a small proportion of patients with low T-score, tibial implant coverage of the proximal tibial bone, or other confounders. Differences in BMD measurement sites could also partly explain differences in the associations found in our study compared with the literature. Previous research has shown a positive correlation between tibial BMD and BMD of the lumbar spine and total hip [21]. The lumbar spine has a higher proportion of trabecular bone and may therefore resemble proximal tibial bone more than total hip BMD, which contains more cortical bone. Yet, sub-analyses in our study using different BMD sites did not show any associations.

### Strengths

The strength of this study is the use of RSA to measure implant migration, a method known for its precision and validity. The precision of RSA in our study was acceptable. The inclusion of a large heterogeneous patient cohort strengthens the generalizability, as it represents the diverse patient group encountered in clinical practice.

### Limitations

Despite the large patient cohort with RSA implant migration data, the number of patients in the subgroups is low. The substantial variability in migration results and patterns indicates that additional variables may influence the observed outcomes more than bone density, or the density at the spine level is not representative of the proximal tibia. The 64 excluded patients who did not have 2 RSA images were older than the total cohort. As expected, older patients are more likely to decline, which could lead to a systematic underestimation of migration. Additionally, a limitation is the absence of data on knee implant alignment, although others reported that 2-year migration did not seem to be associated with alignment [22,23]. Unlike previous studies, which often excluded patients with osteoporosis or bone-related diseases, our study included an unselected patient cohort. The proportion of patients with low T-scores was higher in the cemented groups, reflecting treatment selection bias: surgeons preferentially chose cemented implants for patients with the poorest local bone quality to improve initial fixation. Consequently, the association between low BMD and implant migration might be biased, since the fixation method (cemented/cementless) was influenced by the very factor under study (BMD). Another consideration is the risk of residual confounding, as osteoporosis is associated with comorbidities and other potential risk factors (e.g., smoking, alcohol use, activity level), which could not be accounted for in our study. In contrast, patients with osteoporosis may be receiving antiresorptive treatment, which has a positive effect on implant fixation and acts as a mediator in the causal pathway [24,25]. In our study, no association between T-score groups and tibial implant migration was observed when examining patients without antiresorptive treatment, but this subgroup had wide CIs, possibly relating to a small sample size.

Finally, the results showed some sensitivity to missing data assumptions, particularly in the cementless TKA group, where complete-case analyses yielded differences in point estimates compared with the primary mixed-effects model. However, CIs overlapped, suggesting overall robustness of the findings. When restricting the analyses to 1- and 2-year follow-up, results were consistent with the primary analysis.

### Conclusion

Tibial implant migration was not significantly different between patients with low and normal BMD as defined by T-score groups.

These findings should be interpreted cautiously due to small subgroup sizes and wide confidence intervals. In perspective, further research, on the influence of BMD, fixation, and antiresorptive treatment, is needed to clarify how these factors interact with implant fixation and long-term migration outcomes, and risk of reoperation.

### Supplementary data

Supplementary Tables S1–S6 are available as Supplementary data on the article home page, doi: 10.2340/17453674.2026.46362

## Supplementary Material



## Notes

KNL: conceptualization, data curation, formal analysis, funding, investigation, methodology, project administration, resources, software, validation, visualization, writing—original draft, writing—review and editing. BLL: methodology, supervision, writing—review and editing. SR: conceptualization, analysis, investigation, methodology, resources, supervision, writing—review and editing. RGHHN: methodology, analysis, supervision, writing—review and editing. MS: conceptualization, analysis, funding, investigation, methodology, project administration, funding acquisition, resources, software, supervision, validation, writing—review and editing

The authors would like to thank all the patients and the research support at Aarhus University and Aarhus University Hospital. Thanks also to Kjeld Søballe and Frank Madsen for contributing to establishing the AutoRSA Database. Many thanks to Rikke Mørup for assistance with patient follow-ups, DXA analyses, and RSA analyses. Thanks to Anne Dorthe Riedel for the assistance with RSA analyses. Thanks to the knee surgeons at Aarhus University Hospital for including and operating on the patients.

Handling co-editors: Per Henrik Randsborg and Robin Christensen

*Acta* thanks Hannu T Aro and Marc J Nieuwenhuijse for help with peer review of this manuscript.
