# Supplementary material for: Tibial implant migration of cemented and cementless total and unicompartmental knee arthroplasties in patients with low and normal bone mineral density: a 5-year RSA cohort study of 397 cases
Source: Acta Orthop. 2026 Aug 10;97:523–30. doi: 10.2340/17453674.2026.46362 (PMC13455171; doi:10.2340/17453674.2026.46362)
Supplement: Supplementary file 1 [file ActaO-97-46362-s1.pdf]

Supplementary data

Supplementary Table S1. RSA double examination measurements for the 5 different implant types

Axis	Translations (mm) x	y	z	TT [a]	Rotations (°) x	y	z	TR [b]	MTPM (mm)
Cemented Vanguard TKA, n = 12									
Mean difference	0.00	0.01	–0.02	–0.01	0.01	–0.01	0.03	–0.10	–0.08
SD difference	0.06	0.04	0.10	0.07	0.14	0.30	0.10	0.18	0.16
CR (1.96 * SD difference)	0.11	0.09	0.19	0.14	0.28	0.58	0.19	0.34	0.32
Cementless NexGen Monoblock TKA, n = 51									
Mean difference	0.01	0.01	0.02	–0.01	–0.02	–0.05	–0.02	–0.02	–0.01
SD difference	0.07	0.07	0.25	0.16	0.51	0.31	0.13	0.36	0.20
CR (1.96 * SD difference)	0.14	0.14	0.48	0.31	0.99	0.61	0.26	0.70	0.38
Cemented NexGen Stemmed Precoat TKA, n = 71									
Mean difference	0.00	0.00	0.00	0.00	–0.05	–0.10	–0.00	–0.05	–0.05
SD difference	0.07	0.09	0.19	0.12	0.31	0.38	0.10	0.34	0.24
CR (1.96 * SD difference)	0.15	0.18	0.37	0.24	0.60	0.75	0.19	0.67	0.47
Cemented Oxford UKA, n = 101									
Mean difference	–0.01	–0.01	0.00	–0.01	0.03	0.01	0.02	–0.01	–0.01
SD difference	0.05	0.03	0.11	0.07	0.20	0.18	0.15	0.16	0.09
CR (1.96 * SD difference)	0.10	0.06	0.21	0.14	0.39	0.35	0.29	0.30	0.18
Cementless Oxford UKA, n = 130									
Mean difference	0.00	0.00	0.00	0.00	–0.02	0.00	0.02	0.01	0.00
SD difference	0.03	0.03	0.10	0.08	0.20	0.12	0.15	0.16	0.09
CR (1.96 * SD difference)	0.06	0.07	0.20	0.15	0.38	0.24	0.29	0.31	0.17

The mean difference represents the systematic measurement error. The SD difference represents the random variation (precision). Coefficient of repeatability (CR) (±1.96 * SD difference) represents the expected clinical precision.

[a] Total translation (TT) was calculated using the Pythagorean theorem ($TT = \sqrt{(x^2+y^2+z^2)}$).

[b] Total rotation (TR) was calculated using the Pythagorean theorem ($TR = \sqrt{(x^2+y^2+z^2)}$).

RSA: radiostereometric analysis, MTPM: maximum total point motion, TKA: total knee arthroplasty, UKA: unicompartmental knee arthroplasty, SD: standard deviation.

Supplementary Table S2. Mean translation (Tx, Ty, Tz, Total) (mm) and rotation (Rx, Ry, Rz, Total) (°) with CI in T-score groups for cementless TKA adjusted for BMI and age

Axis and follow-up	Low T-score [a]<br>n = 28<br>mean (CI)	Normal T-score<br>n = 50<br>mean (CI)	Difference<br>mean (CI)
X-axis translation (Tx) mm			
1 year	0.01 (–0.15 to 0.16)	0.00 (–0.11 to 0.12)	0.00 (–0.19 to 0.20)
2 years	–0.03 (–0.17 to 0.12)	–0.02 (–0.12 to 0.09)	–0.01 (–0.19 to 0.17)
5 years	–0.06 (–0.16 to 0.11)	–0.04 (–0.16 to 0.08)	–0.01 (–0.22 to 0.19)
Y-axis translation (Ty), mm			
1 year	–0.06 (–0.19 to 0.07)	–0.01 (–0.11 to 0.08)	–0.05 (–0.21 to 0.11)
2 years	–0.01 (–0.14 to 0.11)	0.00 (–0.10 to 0.09)	–0.01 (–0.17 to 0.15)
5 years	0.03 (–0.09 to 0.16)	0.01 (–0.08 to 0.11)	0.02 (–0.14 to 0.18)
Z-axis translation (Tz,) mm			
1 year	–0.17 (–0.35 to 0.00)	–0.06 (–0.19 to 0.07)	–0.12 (–0.34 to 0.11)
2 years	–0.28 (–0.45 to –0.11)	–0.07 (–0.20 to 0.06)	–0.21 (–0.43 to 0.00)
5 years	–0.31 (–0.52 to –0.10)	–0.10 (–0.26 to 0.06)	–0.21 (–0.48 to 0.06)
Total translation (TT) [b], mm			
1 year	0.59 (0.41 to 0.76)	0.53 (0.39 to 0.66)	0.06 (-0.16 to 0.28)
2 years	0.62 (0.46 to 0.79)	0.51 (0.39 to 0.63)	0.12 (–0.09 to 0.32)
5 years	0.67 (0.47 to 0.88)	0.58 (0.43 to 0.73)	0.10 (–0.16 to 0.35)
X-axis rotation (Rx), °			
1 year	–0.17 (–0.62 to 0.28)	–0.28 (–0.62 to 0.05)	0.12 (–0.45 to 0.68)
2 years	–0.38 (–0.87 to 0.11)	–0.16 (–0.52 to 0.19)	–0.21 (–0.83 to 0.40)
5 years	–0.40 (–0.86 to 0.07)	–0.35 (–0.69 to 0.00)	–0.05 (–0.63 to 0.53)
Y-axis rotation (Ry), °			
1 year	0.22 (–0.06 to 0.50)	0.05 (–0.16 to 0.25)	0.17 (–0.18 to 0.52)
2 years	0.21 (–0.09 to 0.49)	0.02 (–0.19 to 0.23)	0.19 (–0.17 to 0.54)
5 years	0.16 (–0.21 to 0.52)	0.03 (–0.24 to 0.29)	0.13 (–0.32 to 0.58)
Z-axis rotation (Rz), °			
1 year	–0.23 (–0.61 to 0.14)	0.02 (–0.26 to 0.30)	–0.25 (–0.73 to 0.22)
2 years	–0.14 (–0.50 to 0.22)	0.06 (–0.21 to 0.32)	–0.20 (–0.65 to 0.25)
5 years	–0.12 (–0.46 to 0.23)	0.11 (–0.14 to 0.36)	–0.23 (–0.66 to 0.20)
Total rotation (TR) [c], °			
1 year	1.49 (1.09 to 1.90)	1.29 (0.99 to 1.60)	0.20 (–0.30 to 0.71)
2 years	1.49 (1.11 to 1.86)	1.36 (1.09 to 1.64)	0.12 (–0.34 to 0.59)
5 years	0.69 (0.49 to 0.89)	0.60 (0.45 to 0.75)	0.09 (–0.16 to 0.34)

[a] The low T-score group includes patients with T-score ≤ –1.0.
[b] Total translation (TT) was calculated using the Pythagorean theorem (TT= $\sqrt{(x^2+y^2+z^2)}$).
[c] Total rotation (TR) was calculated using the Pythagorean theorem (TR= $\sqrt{(x^2+y^2+z^2)}$).
BMI: body mass index, CI: 95% confidence interval, TKA: total knee arthroplasty.

Supplementary Table S3. Mean translation (Tx, Ty, Tz, Total) (mm) and rotation (Rx, Ry, Rz, Total) (°) with CI in T-score groups for cementless UKA adjusted for BMI and age

Axis and follow-up	Low T-score[a] n = 54 mean (CI)	Normal T-score n = 78 mean (CI)	Difference mean (CI)
X-axis translation (Tx) mm			
1 year	0.10 (0.04 to 0.16)	0.07 (0.02 to 0.12)	0.03 (–0.05 to 0.10)
2 years	0.14 (0.08 to 0.20)	0.13 (0.08 to 0.18)	0.00 (–0.08 to 0.08)
5 years	0.24 (0.17 to 0.30)	0.22 (0.16 to 0.27)	0.02 (–0.07 to 0.11)
Y-axis translation (Ty), mm			
1 year	–0.12 (–0.22 to –0.02)	–0.31 (–0.39 to –0.22)	0.19 (0.05 to 0.32)
2 years	–0.09 (–0.20 to 0.01)	–0.30 (–0.39 to –0.21)	0.20 (0.06 to 0.34)
5 years	–0.06 (–0.18 to 0.05)	–0.28 (–0.37 to –0.19)	0.22 (0.07 to 0.36)
Z-axis translation (Tz,) mm			
1 year	–0.08 (–0.16 to 0.00)	–0.12 (–0.18 to –0.06)	0.03 (–0.07 to 0.14)
2 years	–0.04 (–0.14 to 0.05)	–0.12 (–0.19 to –0.05)	0.08 (–0.04 to 0.19)
5 years	–0.12 (–0.21 to –0.02)	–0.13 (–0.20 to –0.06)	0.01 (–0.11 to 0.13)
Total translation (TT) [b], mm			
1 year	0.44 (0.33 to 0.54)	0.48 (0.40 to 0.55)	–0.04 (–0.17 to 0.09)
2 years	0.47 (0.37 to 0.57)	0.50 (0.42 to 0.58)	–0.03 (–0.16 to 0.10)
5 years	0.55 (0.45 to 0.65)	0.55 (0.48 to 0.63)	0.00 (–0.13 to 0.13)
X-axis rotation (Rx), °			
1 year	–0.58 (–0.95 to –0.21)	–0.91 (–1.18 to –0.63)	0.32 (–0.15 to 0.80)
2 years	–0.65 (–1.03 to –0.27)	–0.90 (–1.18 to –0.62)	0.25 (–0.23 to 0.73)
5 years	–0.69 (–1.09 to –0.29)	–0.92 (–1.21 to –0.62)	0.22 (–0.28 to 0.72)
Y-axis rotation (Ry), °			
1 year	–0.23 (–0.43 to –0.02)	–0.07 (–0.23 to 0.08)	–0.15 (–0.41 to 0.11)
2 years	–0.20 (–0.41 to 0.01)	–0.06 (–0.21 to 0.10)	–0.14 (–0.41 to 0.13)
5 years	–0.21 (–0.43 to 0.00)	–0.09 (–0.25 to 0.07)	–0.12 (–0.40 to 0.15)
Z-axis rotation (Rz), °			
1 year	0.46 (0.12 to 0.80)	0.38 (0.13 to 0.63)	0.08 (–0.36 to 0.51)
2 years	0.31 (–0.03 to 0.65)	0.15 (–0.10 to 0.41)	0.16 (–0.27 to 0.59)
5 years	0.15 (–0.22 to 0.51)	0.06 (–0.21 to 0.32)	0.09 (–0.37 to 0.55)
Total rotation (TR) [c], °			
1 year	1.36 (0.97 to 1.75)	1.58 (1.29 to 1.88)	–0.23 (–0.71 to 0.26)
2 years	1.37 (0.99 to 1.76)	1.59 (1.30 to 1.88)	–0.22 (–0.70 to 0.26)
5 years	0.53 (0.42 to 0.64)	0.52 (0.45 to 0.60)	0.00 (–0.13 to 0.14)

[a– c] See Supplementary Table 2.
BMI: body mass index, CI: 95% confidence interval, UKA: unicompartmental knee arthroplasty.

Supplementary Table S4. Mean translation (Tx, Ty, Tz, Total) (mm) and rotation (Rx, Ry, Rz, Total) (°) with CI in T-score groups for cemented TKA adjusted for BMI and age

Axis and follow-up	Low T-score [a] mean (CI)	Normal T-score mean (CI)	Difference mean (CI)
X-axis translation (Tx) mm			
1 year	0.02 (–0.06 to 0.09)	–0.14 (–0.26 to –0.03)	0.16 (0.02 to 0.30)
2 years	0.00 (–0.08 to 0.08)	–0.15 (–0.28 to –0.02)	0.15 (–0.01 to 0.30)
5 years	–0.02 (–0.11 to 0.07)	–0.13 (–0.27 to 0.00)	0.11 (–0.05 to 0.27)
Y-axis translation (Ty), mm			
1 year	0.12 (0.07 to 0.16)	0.16 (0.09 to 0.23)	–0.04 (–0.13 to 0.04)
2 years	0.15 (0.10 to 0.20)	0.18 (0.11 to 0.26)	–0.03 (–0.12 to 0.05)
5 years	0.18 (0.13 to 0.23)	0.21 (0.13 to 0.28)	–0.03 (–0.12 to 0.06)
Z-axis translation (Tz,) mm			
1 year	–0.04 (–0.15 to 0.08)	–0.26 (–0.44 to –0.08)	0.22 (0.00 to 0.45)
2 years	0.00 (–0.12 to 0.13)	–0.30 (–0.49 to –0.10)	0.30 (0.06 to 0.54)
5 years	0.02 (–0.12 to 0.16)	–0.25 (–0.46 to –0.04)	0.28 (0.02 to 0.53)
Total translation (TT) [b], mm			
1 year	0.46 (0.37 to 0.56)	0.49 (0.34 to 0.64)	–0.03 (–0.21 to 0.15)
2 years	0.49 (0.39 to 0.60)	0.56 (0.40 to 0.72)	–0.07 (–0.26 to 0.13)
5 years	0.52 (0.40 to 0.63)	0.53 (0.36 to 0.71)	–0.02 (–0.23 to 0.19)
X-axis rotation (Rx), °			
1 year	–0.01 (–0.19 to 0.16)	–0.36 (–0.63 to –0.10)	0.35 (0.03 to 0.68)
2 years	0.07 (–0.11 to 0.26)	–0.31 (–0.59 to –0.03)	0.38 (0.04 to 0.72)
5 years	–0.11 (–0.30 to 0.08)	–0.35 (–0.64 to –0.06)	0.24 (–0.12 to 0.59)
Y-axis rotation (Ry), °			
1 year	–0.29 (–0.54 to –0.04)	0.07 (–0.31 to 0.46)	–0.36 (–0.83 to 0.11)
2 years	–0.30 (–0.50 to –0.10)	–0.04 (–0.35 to 0.27)	–0.26 (–0.64 to 0.12)
5 years	–0.25 (–0.50 to –0.01)	0.25 (–0.11 to 0.61)	–0.50 (–0.94 to –0.06)
Z-axis rotation (Rz), °			
1 year	–0.07 (–0.19 to 0.04)	0.21 (0.03 to 0.38)	–0.28 (–0.49 to –0.06)
2 years	–0.08 (–0.20 to 0.05)	0.22 (0.04 to 0.41)	–0.30 (–0.52 to –0.07)
5 years	–0.09 (–0.23 to 0.04)	0.18 (–0.03 to 0.38)	–0.27 (–0.52 to –0.02)
Total rotation (TR) [c], °			
1 year	0.99 (0.76 to 1.21)	0.87 (0.52 to 1.22)	0.11 (–0.31 to 0.53)
2 years	0.98 (0.80 to 1.16)	0.88 (0.61 to 1.15)	0.10 (–0.23 to 0.43)
5 years	0.56 (0.42 to 0.69)	0.55 (0.35 to 0.75)	0.00 (–0.24 to 0.25)

[a–c] See Supplementary Table 2.
BMI: body mass index, CI: 95% confidence interval, TKA: total knee arthroplasty.

Supplementary Table S5. Mean translation (Tx, Ty, Tz, Total) (mm) and rotation (Rx, Ry, Rz, Total) (°) with CI in T-score groups for cemented UKA adjusted for BMI and age

Axis and follow-up	Low T-score [a] mean (CI)	Normal T-score mean (CI)	Difference mean (CI)
X-axis translation (Tx), mm			
1 year	0.12 (0.06 to 0.19)	0.09 (0.03 to 0.14)	0.04 (–0.05 to 0.12)
2 years	0.15 (0.08 to 0.22)	0.11 (0.04 to 0.17)	0.04 (–0.05 to 0.14)
5 years	0.21 (0.12 to 0.31)	0.11 (0.03 to 0.20)	0.10 (–0.02 to 0.23)
Y-axis translation (Ty), mm			
1 year	0.10 (0.02 to 0.18)	0.06 (–0.01 to 0.13)	0.04 (–0.06 to 0.15)
2 years	0.13 (0.03 to 0.21)	0.08 (0.00 to 0.16)	0.05 (–0.08 to 0.17)
5 years	0.13 (0.04 to 0.23)	0.11 (0.02 to 0.19)	0.03 (–0.10 to 0.16)
Z-axis translation (Tz,) mm			
1 year	–0.04 (–0.11 to 0.04)	–0.03 (–0.09 to 0.04)	–0.01 (–0.11 to 0.09)
2 years	–0.05 (–0.12 to 0.02)	–0.04 (–0.10 to 0.02)	–0.01 (–0.11 to 0.08)
5 years	–0.04 (–0.14 to 0.06)	0.00 (–0.09 to 0.09)	–0.04 (–0.17 to 0.10
Total translation (TT) [b], mm			
1 year	0.29 (0.20 to 0.38)	0.35 (0.27 to 0.43)	–0.06 (–0.18 to 0.06)
2 years	0.32 (0.22 to 0.41)	0.38 (0.29 to 0.47)	–0.07 (–0.20 to 0.07)
5 years	0.36 (0.24 to 0.49)	0.48 (0.36 to 0.59)	–0.11 (–0.28 to 0.05)
X-axis rotation (Rx), °			
1 year	–0.17 (–0.31 to –0.03)	–0.23 (–0.36 to –0.11)	0.06 (–0.13 to 0.25)
2 years	–0.22 (–0.40 to –0.05)	–0.31 (–0.47 to –0.16)	0.09 (–0.14 to 0.32)
5 years	–0.28 (–0.47 to –0.10)	–0.27 (–0.43 to –0.10)	–0.02 (–0.27 to 0.23)
Y-axis rotation (Ry), °			
1 year	0.02 (–0.11 to 0.14)	0.06 (–0.05 to 0.18)	–0.05 (–0.22 to 0.13)
2 years	–0.01 (–0.14 to 0.11)	0.06 (–0.05 to 0.17)	–0.07 (–0.24 to 0.10)
5 years	–0.04 (–0.19 to 0.12)	–0.01 (–0.15 to 0.12)	–0.02 (–0.23 to 0.19)
Z-axis rotation (Rz), °			
1 year	–0.26 (–0.43 to –0.10)	–0.19 (–0.34 to –0.04)	–0.08 (–0.30 to 0.15)
2 years	–0.28 (–0.46 to –0.09)	–0.26 (–0.43 to –0.09)	–0.02 (–0.27 to 0.24)
5 years	–0.40 (–0.63 to –0.17)	–0.30 (–0.51 to –0.09)	–0.10 (–0.41 to 0.21)
Total rotation (TR) [c], °			
1 year	0.67 (0.51 to 0.82)	0.80 (0.66 to 0.94)	–0.14 (–0.35 to 0.08)
2 years	0.67 (0.49 to 0.85)	0.94 (0.77 to 1.11)	–0.27 (–0.52 to –0.02)
5 years	0.35 (0.22 to 0.49)	0.49 (0.37 to 0.62)	–0.14 (–0.33 to 0.04)

[a–c] See Supplementary Table 2.

BMI: body mass index, CI: 95% confidence interval, UKA: unicompartmental knee arthroplasty.

Supplementary Table S6. Number of patients with continuous migration. Values are count

Factor	Continuous migration between 1 and 2 years (> 0.2 mm)	Continuous migration between 2 and 5 years (> 0.1 mm /year)
Cementless TKA (yes/no)	17 / 54	27 / 51
Cementless UKA (yes/no)	15 / 107	45 / 87
Cemented TKA (yes/no)	21 / 51	46 / 37
Cemented UKA (yes/no)	13 / 85	28 / 76

TKA: total knee arthroplasty, UKA: unicompartmental knee arthroplasty.
